# A Personal Statement

**Published:** 1914-06

**Authors:** F. E. Daniel


### EDITORIAL DEPARTMENT.

**DR. R. H. L. BIBB, Associate Editor**

DEPARTMENTS { EUGENICS: Drs. M. Duggan and T. Y. Hull.

WOMAN'S DEPARTMENT: Mrs. F. E. Daniel.

### A Personal Statement.

Most readers of the TEXAS MEDICAL JOURNAL already know of the passing, on May 14th, of my husband, Dr. F. E. Daniel, the founder and for nearly thirty years the editor and publisher of the JOURNAL. Many of you will recall his feeble condition at the State meeting in San Antonio last year. He continued to grow more feeble until the end, which was peaceful and beautiful, if such can be said of death.

For many months he realized that his life's work was nearing the end, and many times he expressed a desire to join his comrades on the other side, of whom he often spoke during the weeks and days of gradual dissolution. When he talked of the dear ones gone before his face would brighten in anticipation of the meeting.

He delighted to quote from St. Paul: "I have fought the good fight; I have kept the faith," and he would always add: "The medical faith." And truly he had, for no man ever lived who had loftier ideals, or endeavored harder to impress those ideals upon other members of the profession. They were a part of his very life, and he could no more have helped teaching and preaching them than he could have overcome his innate Virginia chivalry.

He devoted the best of his life to helping humanity, building up higher standards of conception for coming generations, because he loved humanity.

The TEXAS MEDICAL JOURNAL was the beloved child of his brain, the embodiment of his professional ideals, and is a fitting memorial to him. It was his desire that it should live after him and that its usefulness should increase with its age. For several years past I was closely associated with him in the work, and I have endeavored by close study to prepare myself to continue its publication successfully. I shall do so not only because it is necessary, but because I like the work and believe that, with the assistance of many friends, I can make it helpful. While I realize the difficulties of the task before me, I do not doubt for a moment that I shall succeed. Already I have the voluntary offer of editorial help from a number of the best and most progressive men in the State, who will give the readers of the Journal the very best that is in them, and others will no doubt be glad to aid me in this way when their attention is called to it. Articles will be contributed from leaders in the medical profession in the State and out of it, and events of interest to the profession at home and abroad will be duly chronicled.

The Journal will remain, as it has always been, independent; will stand for the highest ethical ideals, and will be free to commend or criticize as occasion may demand.

Please do not think that because the Journal is under the management of a woman that it is to be a milk-and-water publication. Those of you who know me, know that I believe that a thing worth doing is worth doing well; that in principle and by training I believe in striving for the best, and that I shall not be satisfied unless the Journal can furnish intellectual food for those in the profession who are ready for strong diet.

I am young, strong and ambitious, with a restless love for work, and shall give my entire time and attention to the improvement of the Journal, and to better equipping myself for my life work.

I feel that only the best is good enough for the readers of the "Red Back," most of whom are progressive students, who subscribe for medical publications for the help to be obtained from them. I shall make an earnest effort to keep abreast of all forward movements in the profession, and to make the Journal, in every issue, a source of profit to every reader.

The many assurances of sympathy, confidence and readiness on the part of medical friends and advertisers to assist me in every way possible have made me feel a keen desire to measure up to the highest expectation of these loyal supporters. I must succeed, if for no other reason, because of their unbounded and unfaltering trust in me.

I appreciate, more than words can tell, every letter and word of encouragement that has come to me in this hour of grief, and for lack of time to answer each of them by personal letter I wish now to thank every one for expressions of comfort and encouragement.

While appreciative of sympathy, I do not desire business because you may sympathize with me. I want you to read every number of the Journal carefully, and if, at the end of the year, you feel that you have not received full value for your subscription, I shall gladly refund the money paid me, and thank you for your honesty in telling me that the Journal was not up to your demands. If you find it interesting, I shall appreciate a line from you to that effect—it will encourage me.

Respectfully,

MRS. F. E. DANIEL.

